# Online citizen science with the Zooniverse for analysis of biological volumetric data

**DOI:** 10.1007/s00418-023-02204-6

**Published:** 2023-06-07

**Authors:** Patricia Smith, Oliver N. F. King, Avery Pennington, Win Tun, Mark Basham, Martin L. Jones, Lucy M. Collinson, Michele C. Darrow, Helen Spiers

**Affiliations:** 1 The Rosalind Franklin Institute, Harwell Campus, Fermi Avenue, Didcot, OX11 0FA UK; 2 Diamond Light Source, Harwell Campus, Fermi Avenue, Didcot, OX11 0DE UK; 3 The Francis Crick Institute, London, NW1 1AT UK

**Keywords:** Volume electron microscopy, Citizen science, Crowdsourcing, Segmentation, Image processing, Machine learning, Cell biology, Cellular imaging, Image classification

## Abstract

Public participation in research, also known as citizen science, is being increasingly adopted for the analysis of biological volumetric data. Researchers working in this domain are applying online citizen science as a scalable distributed data analysis approach, with recent research demonstrating that non-experts can productively contribute to tasks such as the segmentation of organelles in volume electron microscopy data. This, alongside the growing challenge to rapidly process the large amounts of biological volumetric data now routinely produced, means there is increasing interest within the research community to apply online citizen science for the analysis of data in this context. Here, we synthesise core methodological principles and practices for applying citizen science for analysis of biological volumetric data. We collate and share the knowledge and experience of multiple research teams who have applied online citizen science for the analysis of volumetric biological data using the Zooniverse platform (www.zooniverse.org). We hope this provides inspiration and practical guidance regarding how contributor effort via online citizen science may be usefully applied in this domain.

## Introduction

Terabyte-size biological volumetric datasets, composed of mesoscale details of cells or tissues, are now routinely produced (Denk and Horstmann 2004; Micheva and Smith 2007; Xu et al. 2017; Scheffer et al. 2020; Peddie et al. 2022). These data often require human 'annotation'; defined as adding meaning through placing marks, drawings, or noting information (see Table 1 for all 'definitions'). Annotation allows downstream analysis through enabling features of interest to be quantitatively examined, for example, through assessing the number, size, shape, location or spatial relationships of objects. However, manual annotation can be a challenging, time-consuming and subjective process, which consequently limits the reliability, scalability and reproducibility of this approach.

**Table 1:** Glossary of key terms related to building a volumetric biological image analysis citizen science project with the Zooniverse

Term	Definition
Aggregation	Combining multiple classifications associated with a subject to form consensus.
Annotation	Adding meaning to image data through placing marks, drawing or noting information about the content of the image.
Classification	All data associated with a contributor response to a workflow for an individual subject.
Classification interface	Online interface where contributors complete the project workflow.
Contributor	Individual who submits data through completing the project workflow. Encompasses volunteers, researchers, students, etc.
Field guide	A place to store general project information relevant to completing the project workflow(s). It is accessible to contributors as a clickable tab on the right-hand side of the classification interface.
Flipbook	Multiple images can be displayed as a single subject using the Flipbook functionality.
Help text	A pop-up window available in the classification interface when contributors click 'Need some help?'.
Launch	Commencing data collection by sharing a Zooniverse project with contributors.
Manifest	A .csv or .tsv file that provides information about project subjects.
Project builder	Online interface for creating a citizen science project with the Zooniverse (www.zooniverse.org/lab).
Project lead	Individual building a Zooniverse project.
Retirement limit	Number of classifications required per subject.
Segmentation	Process of assigning pixels of a digital image into separate regions.
Subject	A unit of data to be classified. Subjects are presented to a contributor within the classification interface. Subjects can be images, videos, gifs, audio, numerical data, text.
Talk	Discussion forum associated with each project. Default and customised ‘talk boards’ can be created by the project lead.
Task	A unit of work associated with a subject. Can be of the class ‘question’, ‘drawing’, ‘text’ or ‘survey’.
Tutorial	Step-by-step introduction to the project task(s). Displays as a pop-up the first time a volunteer visits a project and remains accessible as a tab within the classification interface thereafter.
Volumetric imaging data	Three-dimensional data from e.g., electron or X-ray imaging platforms.
Workflow	Sequence of tasks completed by a contributor for a subject.
Zooniverse	An open, web-based platform for crowdsourced classification of data.

As the size and number of volumetric datasets continue to grow, novel analytical approaches are increasingly being developed by the research community to provide the annotations needed for downstream quantitative analyses. Recent efforts have led to successful application of machine learning approaches for the analysis of biological volumetric data in several contexts, including 'segmentation' of nuclei and nucleoli in cancer biopsies (Machireddy et al. 2021), plasma membrane of HeLa cells (Karabağ et al. 2021), instance segmentation of mitochondria (Conrad and Narayan 2023) and diverse organelle classes from multiple cell types (Heinrich et al. 2020, 2021). However, automated analytical approaches often fail to generalise well, and so hand-labelled data remains needed for quantitative analysis and model retraining.

To accelerate the process of manual annotation, research teams are increasingly adopting novel approaches, such as working with ‘citizen scientists’ to label large datasets. ‘Citizen science’ is a term that encompasses a diverse and growing set of research practices, from donating idle computer time to research projects (Anderson 2004), self-reporting the symptoms of illnesses, such as coronavirus disease (COVID) (Varsavsky et al. 2021), and working in community biology labs (Landrain et al. 2013), to analysing protein structures online (Cooper et al. 2010). Yet, despite this diversity, citizen science may be understood simply as involvement of (presumed) non-professionals in research (Bonney 1996).

Multiple projects to date have demonstrated that citizen scientists can be effectively engaged in a wide variety of biological image analysis tasks, such as the tracing of connected neurons through electron microscopy (EM) data in Eyewire (Kim et al. 2014), clicking on protein particles in cryo-EM data in Microscopy Masters (Bruggemann et al. 2018) and scoring tumour markers in pathology samples in Cell Slider (Dos Reis et al. 2015), amongst others (Smittenaar et al. 2018; Ørting et al. 2019; Benhajali et al. 2020; Fisch et al. 2021; Fowler et al. 2022). The efficacy of applying online citizen science for the analysis of biological image data, both as an avenue for producing novel research and as a powerful tool for public engagement, has led to a rise in interest from the biological volumetric imaging research community in applying this methodology.

However, there is currently little guidance to support research teams wishing to venture into the citizen science arena. Navigating the development of novel citizen science projects can be challenging, particularly as several options exist for developing and launching an online citizen science project, from creating a stand-alone, fully customised project to enable a specific task [e.g. 'Eyewire' Kim et al. (2014)], incorporating citizen science into online computer games [e.g. Project Discovery in EVE Online (Sullivan et al. 2018)], to using a platform with a generic project building solution such as The Zooniverse [e.g. 'Microscopy Masters' (Bruggemann et al. 2018), 'Etch A Cell' (Spiers et al. 2021)]. Although not traditionally considered to be citizen science (Cohn 2008), pay-for-service options are also available, including annotation platforms such as Quantius (Hughes et al. 2018) and WebKnossos (Boergens et al. 2017) and micro-task services such as Amazon Mechanical Turk (https://www.mturk.com/) (Rädsch et al. 2023).

In this manuscript, we collate and present the expertise of two research teams running complementary collections of citizen science projects for the analysis of biological volumetric data; 'Etch A Cell' and 'Science Scribbler'. The 'Etch A Cell' online citizen science projects (also known collectively as 'The Etchiverse') were conceived in 2016 at The Francis Crick Institute in collaboration with the University of Oxford. Following the success of the first 'Etch A Cell' project (titled 'Etch A Cell') which demonstrated that online citizen scientists can contribute high quality freehand segmentations of the nuclear envelope (NE) (Spiers et al. 2021), multiple additional 'Etch A Cell' projects have been developed and launched. To date, all 'Etch A Cell' projects have involved freehand segmentation of volumetric EM data, however, each novel project has focussed on the segmentation of a different organelle class. The 'Science Scribbler' projects were started in 2018 by a team working at Diamond Light Source, the UK's national synchrotron facility, also in collaboration with the University of Oxford. The first project asked online citizen scientists to annotate the shape and position of organelles in an image volume collected using an X-ray tomography technique (Harkiolaki et al. 2018). Volumetric EM data has formed the basis of subsequent projects which include collecting annotations on the location and life stage of virus particles in an infected cell and the position of mitochondria in image volumes of human placental tissue. Collectively, the 'Etch A Cell' and 'Science Scribbler' research teams have built and launched nine projects using biological volumetric imaging data (Table 2).

**Table 2:** Further resources relevant to developing a Zooniverse citizen science project

Resource	Location
Project building	
Zooniverse website	www.zooniverse.org
Project builder interface	www.zooniverse.org/lab
Project building guidance	help.zooniverse.org
Glossary of Zooniverse terms	help.zooniverse.org/getting-started/glossary
Data handling	
Data digging repository	github.com/Zooniverse/Data-digging
Aggregation code	github.com/zooniverse/aggregation-for-caesar
Aggregation code documentation	aggregation-caesar.zooniverse.org/docs
Panoptes (Zooniverse API)	github.com/zooniverse/Panoptes
Panoptes python client	github.com/zooniverse/panoptes-python-client
Panoptes command-line interface	github.com/zooniverse/panoptes-cli/blob/master/README.md
Connecting	
Zooniverse wide Talk forum	www.zooniverse.org/talk
Zooniverse Twitter	@the_zooniverse
Zooniverse Facebook	www.facebook.com/therealzooniverse
Zooniverse YouTube	@the_zooniverse
Zooniverse Instagram	@the.zooniverse
Etch A Cell resources	
Etch A Cell project collection	www.zooniverse.org/organizations/h-spiers/etch-a-cell-colouring-in-cells-for-science
Etch A Cell	www.zooniverse.org/projects/h-spiers/etch-a-cell
Etch A Cell – Powerhouse Hunt	www.zooniverse.org/projects/h-spiers/etch-a-cell-powerhouse-hunt
Etch A Cell – VR	www.zooniverse.org/projects/h-spiers/etch-a-cell-vr
Etch A Cell – ER	www.zooniverse.org/projects/h-spiers/etch-a-cell-er
Etch A Cell – Fat checker	www.zooniverse.org/projects/dwright04/etch-a-cell-fat-checker
Etch A Cell – Fat checker round 2	www.zooniverse.org/projects/suhailalnahari/etch-a-cell-fat-checker-round-2
Etch A Cell Twitter	@EtchACell
Aggregation code	www.github.com/FrancisCrickInstitute/Etch-a-Cell-Nuclear-Envelope
Science Scribbler resources	
Science Scribbler project collection	www.zooniverse.org/organizations/smith-p/science-scribbler
Science Scribbler	www.zooniverse.org/projects/msbrhonclif/science-scribbler
Science Scribbler: Virus Factory	www.zooniverse.org/projects/markbasham/science-scribbler-virus-factory
Science Scribbler: Placenta Profiles	www.zooniverse.org/projects/msbrhonclif/science-scribbler-placenta-profiles
Science Scribbler Twitter	@ScienceScribbl1
Science Scribbler YouTube	@sciencescribbler
Other	
Zooniverse publications	www.zooniverse.org/about/publications
Downloadable materials	www.zooniverse.org/about/resources
Daily Zooniverse	daily.zooniverse.org
Zooniverse blog	blog.zooniverse.org

Here, we synthesise and share the lessons learned in leading this body of work to provide guidance to support other research teams wishing to develop similar citizen science projects.

## Main

### Overview of the Zooniverse online citizen science project lifecycle

**Fig. 1:**
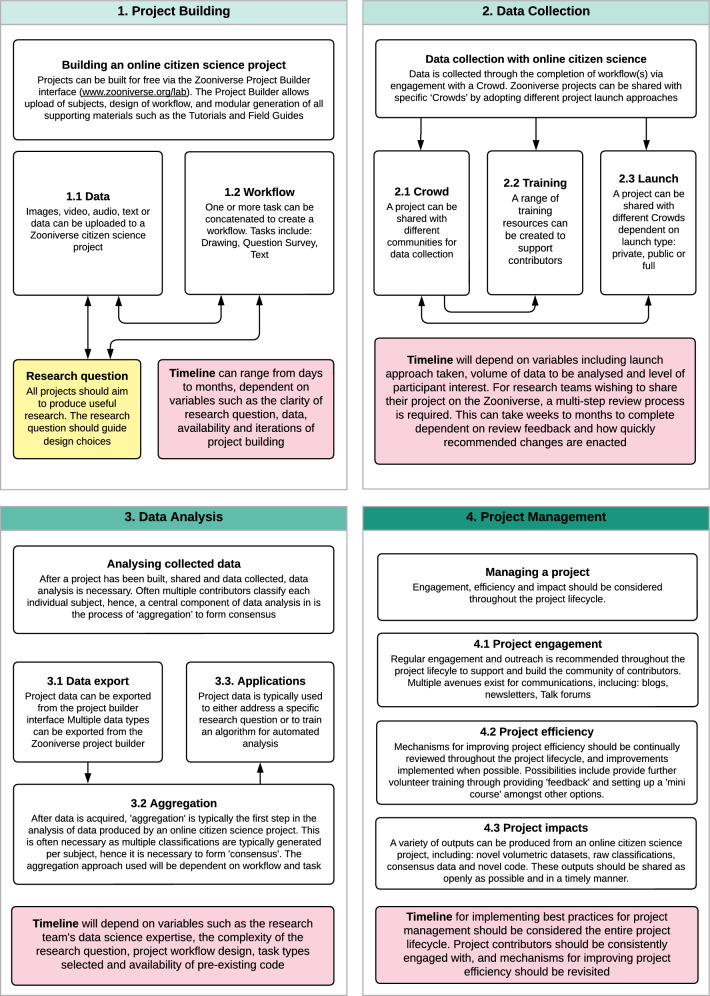
Overview of building a project with the Zooniverse. The four core lifecycle stages and key considerations are outlined

**Fig. 2:**
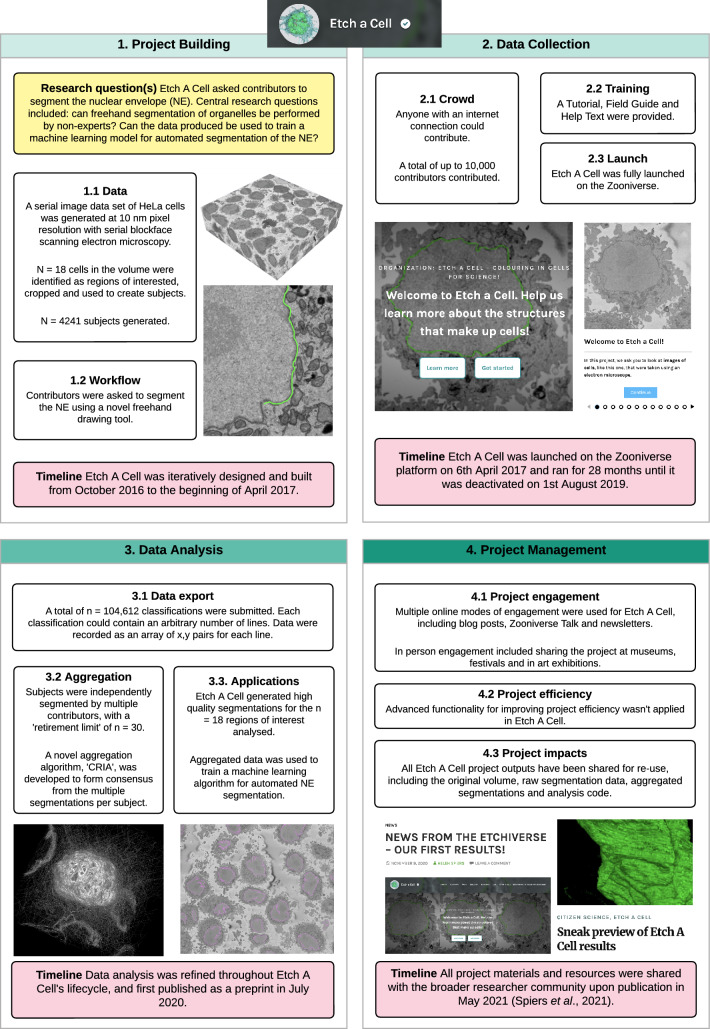
Project lifecycle of 'Etch A Cell'. This figure provides an overview of the key design choices and decisions made for the first 'Etch A Cell' project, for the segmentation of the nuclear envelope (Spiers et al. 2021)

**Fig. 3:**
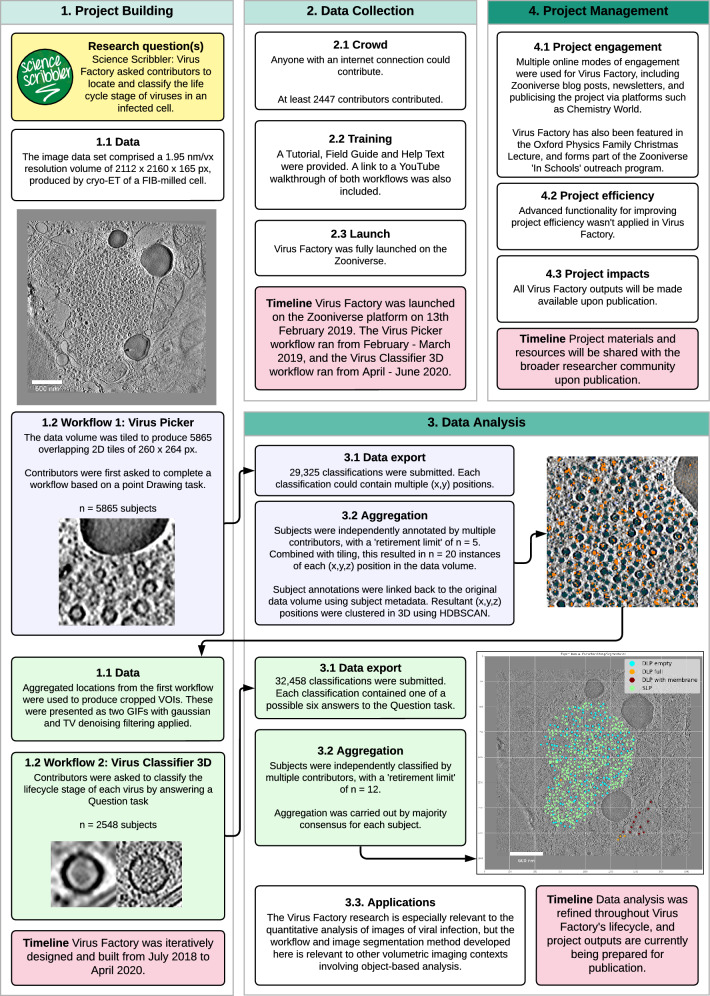
Project lifecycle of 'Science Scribbler: Virus Factory'. This figure provides an overview of the key design choices and decisions made for 'Science Scribbler: Virus Factory', for the localisation and classification of virus particles

The lifecycle of an online citizen science project with the Zooniverse platform (Fig. 1, 2 and 3) can be defined by four stages; project building: encompassing the conception and refining of a research question to be addressed through online citizen science, and the iterative development and building of a project ("Project building"), data collection: sharing of a project with a community of citizen scientists to classify project data ("Data collection"), data analysis: the formation of consensus from collected data ("Data analysis") and finally, project management: engagement and communication with relevant audiences ("Project management").

The project lifecycle is presented in this ordered manner for simplicity, and because there is some linearity in sequence between these four project stages. For instance, it is not possible to collect data until a project has been built. However, there is a large degree of interaction and concurrence between these stages. For example, it is possible, and advisable, to generate and analyse data produced by a pilot version of an online citizen science project, to enable iterative and evidence-based refinement of project design, prior to project 'launch'. We will outline the range of possibilities, considerations and recommendations in relation to the ordering of and interrelation of these four stages where relevant throughout this paper.

The timelines associated with these four lifecycle stages can vary greatly between projects (Fig. 1, Figs. 2 and 3). Multiple variables influence timelines e.g. the time required to build a novel project will be influenced by the complexity of the research question and the expertise of the research team. The time to complete data collection will depend on a range of factors, including the amount of data to be classified, the length and complexity of the project ‘workflow’, and the engagement of the ‘contributor’ community (Spiers et al. 2019). While a number of these variables are within the direct control and influence of the research team, others, such as project popularity, can be beyond control. Consequently, it can be challenging to advise regarding expectations in relation to timelines. However, there are a number of practical suggestions to expedite the project lifecycle and generate outputs quickly, which are noted where relevant below.

The Zooniverse platform encompasses a broad range of functionalities; however, we shall focus here on the components relevant to the design and implementation of online citizen science projects for the annotation of biological ‘volumetric imaging data’. Specific attention will be paid to the features used for ‘Etch A Cell’ and ‘Science Scribbler’ project collections.

## Project building

All citizen science projects presented here were built with a modular online interface called the ‘Project Builder’ (www.zooniverse.org/lab, Table 1) (Trouille et al. 2017), provided by the Zooniverse platform (Table 2). Two components are core to building an online citizen science project for biological volumetric data analysis: what data will be analysed and what series of ‘tasks’ will be required to annotate the data sufficiently for further analysis. Through careful consideration and combination of these components a broad array of online citizen science projects may be built to address a variety of research questions.

### Data

The data to be analysed is arguably the most critical component of an online citizen science project, as the features and focus-matter of the data dictate what research questions can be asked and how. Moreover, variables such as the size of the dataset will impact the number of contributor ‘classifications’ sought, whereas features such as data clarity and quality can influence project design and subsequent contributor experience, and hence impact the amount and quality of classification data returned by the contributing community. Two broad themes require consideration when preparing data for inclusion in an online citizen science project. Data must meet the technical requirements of the citizen science platform, and data should be prepared and presented in an optimal way to support project contributors in the effective completion of the project tasks(s).

#### Data type and metadata

The Zooniverse platform can display multiple types of data for annotation via an online interface (‘classification interface’). Several data types can be presented to contributors for classification (as detailed in the Zooniverse Project Builder). Of these, image, video and GIF data types have proven the most appropriate for the presentation of biological volumetric imaging data. To prevent using a large amount of a contributor’s data bandwidth, file upload size should be as small as possible, with file sizes recommended to be below 600 KB and restricted to a maximum size of 1 MB. Project data, consisting of 'subjects' and a 'manifest' file are uploaded for inclusion in a project either directly via a Graphical User Interface (GUI) within the Zooniverse Project Builder, or via command line (Table 2). Additional metadata fields, as defined by the research team, can be uploaded within the manifest file, with this metadata serving two primary functions; to provide the contributors with further information about the subjects via the classification interface, and to associate project classification data with the original data. As a minimum, we recommend a manifest file should have information linking each subject to an (*x*, *y*, *z*) coordinate within the original data volume.

#### Data acquisition and preparation

Multiple aspects of data acquisition and pre-processing can be modified to alter how and what data is presented to contributors. The critical importance of data preparation and presentation should not be understated, as project data intimately underlies contributor experience, which can impact engagement and influence the number, and quality, of classifications received. Clearly, this can significantly impact downstream analysis and, ultimately, the outputs generated by an online citizen science project ("Project impacts").

The project building pipeline should start with acquisition of high quality data optimised for the intended task. The feature of interest should be as easily identifiable as possible. Where relevant and feasible, markers can be used to make the feature of interest more recognisable. For instance, in 'Etch A Cell - ER', contributors were asked to segment the endoplasmic reticulum. To make this task simpler, data were used where the endoplasmic reticulum had been selectively stained, making this feature of interest clearer against the image background and easier to distinguish from other organelle classes. Following data acquisition, effective data pre-processing can be applied to emphasise the feature of interest. The feature of interest may be made clearer through approaches such as reducing noise or increasing contrast, whereas binning can provide a mechanism to reduce high-resolution noise.

Pre-existing analytical approaches can be applied to augment the data, to provide additional context, clarity and information to contributors and to pre-select appropriate data for analysis. In ‘Etch A Cell – VR’ (a project in which contributors are segmenting multiple organelle classes in a single dataset) we applied our pre-existing machine learning algorithm developed for the automatic segmentation of the NE (Spiers et al. 2021) to segment this structure, to provide further cellular context to inform and guide contributors, and reduce the contributor effort required.

Following acquisition of appropriate data, pre-processing and augmentation and the identification of region(s) of interest (ROIs), the research team will need to also consider the field of view (FOV) of the individual subjects to be presented to contributors within the Zooniverse classification interface. Typically, a compromise is struck between providing sufficiently broad visual context to perform the task, while restricting the FOV to a suitable size (e.g. appropriate zoom level, reasonable number of features of interest to be annotated per image, etc.). In the first 'Etch A Cell' project to study the NE, the FOV was restricted to include ROIs containing single whole cells. This provided contributors with sufficient context to segment the NE within the target cell, while preventing confusion that may have arisen if presenting the whole FOV for segmentation, which contained dozens of cells. In ‘Science Scribbler: Placenta Profiles’, where multiple cell types are present, the data were pre-segmented to only display the cell type of interest, and the data cropped and tiled to create a reasonably sized FOV to present to project contributors.

**Fig. 4:**
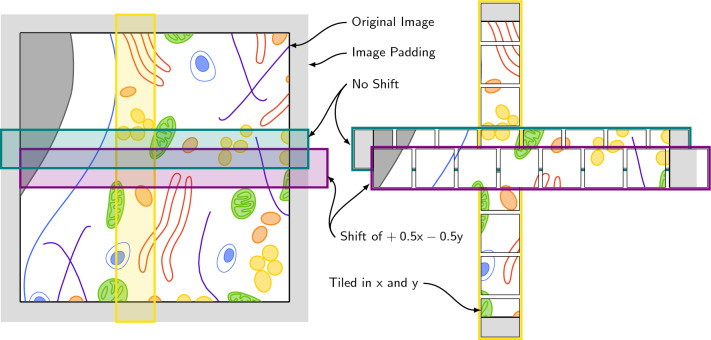
Biological volumetric data can be tiled to generate subjects for online citizen science. Tiling may be necessary to reduce the field of view to a reasonable size to be presented to contributors for classification within a project. Here, we show an example tiling schema. To avoid reduced annotation quality at tile edges, original images are padded, and tiles are produced with an overlap in $x$ and $y$ of 50% of the tile width. This ensures full coverage of the original image, whereby any edge or corner features in one tile will be represented in the centre of an adjacent tile. Tiles are produced in both $x$ (blue) and $y$ (yellow). Overlap can be in $x$,$y$ or both $x$ and $y$ (purple). This example tiling schema would result in each pixel appearing in more than one subject at different locations within the frame. Due to the increased coverage that results from tiling data, it may be possible to reduce the retirement limit

Appropriate FOVs may be generated through selecting specific ROIs from within the data or through tiling the entire volume to a suitably dimensioned FOV. In both ‘Science Scribbler—Placenta Profiles’ and ‘Etch A Cell—Powerhouse Hunt’, data were tiled for upload as the entire volume was considered to be of interest in each project. In this case, it is important to consider whether the data are appropriately sampled e.g. some features of interest may cross tile boundaries, which can impact how and whether they are annotated. This challenge can be addressed by generating overlapping tiles (Fig. 4), e.g. an overlap of 50% in *x* and *y* will ensure cross-boundary objects in one subject are represented centrally in another. To ensure equal representation across the entire dataset, padding can be added to the edges of the data in *x* and *y*. When tiling data, care must be taken to determine how many times each pixel will be represented within the subject data created, and the number of contributors required to annotate each individual subject should be adjusted appropriately to ensure efficient application of contributor time.

#### Presenting volumetric data

**Fig. 5:**
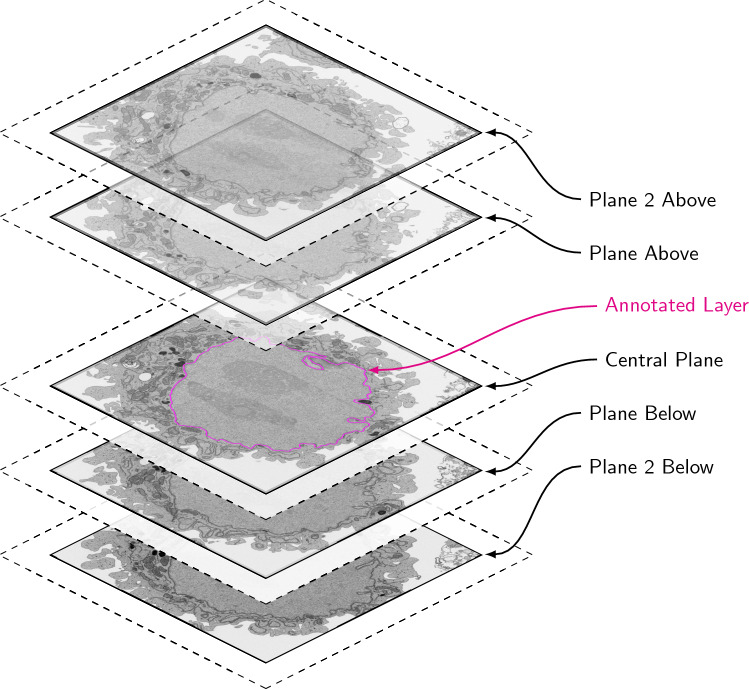
Multiple subjects can be presented as an interactable flipbook to provide 3D context when needed for a given task. In the first ‘Etch A Cell’ project, contributors were presented a stack of five images with FOV cropped to show a single cell. Contributors were asked to use the freehand drawing tool to annotate the nuclear envelope in the central plane of the image stack. Two slices above and below the annotation plane were included to aid in the distinction of nuclear envelope from other features within the images

When working with volumetric datasets, a unique challenge can be effectively presenting three-dimensional (3D) data to contributors. First, it should be considered whether 3D context is needed to effectively answer the research question, given the crowd sought and the project workflow design. In the first ‘Science Scribbler’ project, subjects were individual 2D images, since 3D information was not considered necessary for the task. If 3D context is important, it is possible to present 3D data with the Zooniverse through using stacked 2D images as a ‘flipbook’, video or GIF. In ‘Etch A Cell’ multiple images were displayed as a single subject, using flipbook functionality to provide additional 3D context for the segmentation of the NE (Fig. 5), as described by Spiers et al. (2021). In one workflow of 'Science Scribbler: Virus Factory', two GIFs were displayed side-by-side, showing the same data volume with either Gaussian filtering or total variation denoising filtering applied (Fig. 3). In addition to providing 3D visual context, this presentation of data helped mitigate ambiguity arising from the small scale of the individual virus particles and high image noise, and showing both filtering options was found to help aid in decision making during classification.

Finally, how to sample the data volume in the *z* direction needs to be considered, regardless of whether 2D images or flipbooks are being used. If every slice of the data is included, complete volume coverage will be achieved but the resulting large number of images will take longer for the contributors to annotate, potentially leading to demotivation as the project workflow progresses slowly. However, if every slice is not sampled, this will lead to a distortion of scale in the *z* direction which will then have to be accounted for in downstream data analysis. As seen in ‘Etch A Cell’, there can also be considerations around the quality of annotations near the beginning and end of the stack, where there can be reduced cellular context and greater visual ambiguity due to changes in cellular structure (Spiers et al. 2021). Similarly, in ‘Science Scribbler’, missing wedge artefacts, caused by the limited angle of acquisition of some imaging systems (Bartesaghi et al. 2008), can result in reduced information at the tops and bottoms of some objects. Including orthogonal slices through the same volume may resolve such issues.

### Workflow

The workflow refers to a task, or series of tasks, completed by a contributor in relation to a project subject. The Zooniverse provides a number of standard task types, including ‘drawing’, ‘question’, ‘survey’, and ‘text’, which can be concatenated together to form a complex workflow. In many cases, the information needed to answer the research question can be gathered in multiple ways. We advise that workflow design choices should be guided by the principle of creating the simplest workflow for the contributors.

Of these tasks, the ‘drawing’ and ‘question’ task types have found most utility for the analysis of biological volumetric imaging data to date on the Zooniverse platform. The ‘drawing’ task encompasses a range of marking options (including circles, points, polygons, triangles and freehand lines) that can be included in a workflow to enable contributors to designate features of interest within a subject through adding relevant marks. For example, in ‘Etch A Cell – Powerhouse Hunt’ contributors were asked to trace over the outline of any mitochondria in the subject, while in ‘Science Scribbler’, contributors were asked to mark the centre of any organelle in the image and outline them with an ellipse tool.

The ‘question’ task presents a question and a selection of possible pre-written answers for the contributors to select from. In the context of volumetric imaging data, the ‘question’ task has been applied to gather further information about the objects identified within the subjects. In ‘Science Scribbler: Virus Factory’, two workflows were developed to generate the required project data (Fig. 3). The first workflow asked contributors to use a ‘drawing’ tool to mark the location of virus particles. Following outlier removal and clustering of the outputs from the first workflow, the second workflow used a ‘question’ task to ask contributors to classify the lifecycle stage of each marked virus.

A central concept in online citizen science is that multiple individuals can contribute classifications to the same subject, and that collectively these classifications can produce high quality data for further analysis ("Data analysis"). The number of classifications required per subject is termed the ‘retirement limit’, and is modifiable for each workflow. When determining the retirement limit, the complexity of the task should be taken into account, and ideally pilot data should be collected and analysed to determine a reasonable number of classifications to seek per subject prior to project launch ("Data collection"). Our broad guidance is that the retirement limit should be as low as possible to optimise contributor effort, while ensuring the data produced remains sufficiently high quality for downstream analysis.

## Data collection

Project classification data is collected through the completion of project workflow(s) via engagement with a community of contributors. Zooniverse projects can be shared with specific ‘crowds’ through adopting different project launch approaches. This section describes common rationales for engaging with different crowds, the training that may be provided for these differing communities, before outlining the steps required for different launch approaches.

### Crowd

Citizen science involves a community of individuals (the ‘crowd’) collectively classifying project data. The central decision in this context is whether a project will be shared privately with a defined community, such as professional researchers, a patient group or students, etc., or whether a project will be made publicly accessible to all. The crowd sought will relate to a range of project considerations. The typical rationale for making a project publicly accessible is a desire to engage with as many individuals as possible to quickly analyse a large dataset and to maximise public engagement. The vast majority of ‘Etch A Cell’ and ‘Science Scribbler’ projects have been shared in this way.

The rationale for working with private communities can be more varied. A project may be shared privately with a defined community of known ability if particularly high-quality data is sought, e.g. in ‘Science Scribbler: Placenta Profiles’, a small amount of data was shared privately with experts to generate high quality examples to be used for contributor training. Other reasons to share a dataset with a private community can include if the dataset to be analysed is small and the classifications can be completed by engagement of a small, private community, such as a lab group of researchers or a classroom of students. This approach was adopted by 'Etch A Cell—mitochondria mega mix'; this project shared a small amount of data with students through a private project (Conrad and Narayan 2023). This expedited project completion and data analysis—since the review process necessary for publicly launched Zooniverse projects was not required. Finally, although not yet applied by the ‘Etch A Cell’ or ‘Science Scribbler’ projects, combined approaches of working with both private and public crowds can provide even more study design flexibility. Through taking a combinatorial approach it can be possible to create complex, multi-step workflows with different crowds contributing at different stages of the data analysis pipeline.

### Training

Regardless of the crowd engaged, training resources will likely be required to provide contributors with sufficient knowledge to complete the project workflow(s). Multiple features are available on the Zooniverse platform to provide contributors with the required information.

Training resources include the 'tutorial', 'field guide' and 'help text'. Each can be customised to provide information specific to the project, workflow(s) and subject matter. All three training resources can incorporate text, videos, images and hyperlinks. The training resources are hierarchical: the field guide is universal across the entire project, tutorials are specific to each workflow, and help text features are specific to tasks within each workflow. Collective utilisation of these tools allows for comprehensive training with built-in redundancy, so that contributors may find assistance with the correct specificity and detail.

In addition to the resources designed to train contributors to effectively complete the project workflow(s), there are static 'about' pages that provide a place for research teams to share further project information. Research teams are encouraged to provide information related to the background and research motivations of the project (‘research’ page), team member information (‘team’ page), project results and findings (‘results’ page), information about any educational efforts (‘education’ page) and frequently asked questions (‘FAQ’ page).

Across all project resources, it is best practice to provide information using plain language with as little jargon as possible. Terminology should be consistent, and text should be as brief as possible while conveying all necessary information. We recommend the training resources include both positive and negative examples and common troubleshooting information. All materials should be created with the target contributor audience in mind ("Crowd"), e.g. while explanations and examples will be necessary for both the general public and expert, the level of detail required will be different.

### Launch

Project launch refers to sharing a project with contributors. There are multiple ways an online citizen science project can be launched with the Zooniverse platform ('private launch', ‘public launch’ and ‘full launch’), and the mechanism used provides a means to share the project with different crowds.

#### Private launch

‘Private launch’ is a mode of sharing a Zooniverse project with specific named contributors. This provides a mechanism for controlling who can see and contribute to a project. Applications of privately distributing an online citizen science project include sharing a project with a specific community of contributors and collaborators to solicit project feedback prior to sharing more broadly via a Public or Full Launch. Alternatively, for a variety of reasons, a research team may wish to only collect annotations from a specific community of known contributors. For example, in the context of biological volumetric data annotation, privately launching a Zooniverse project has been applied to gather ‘expert annotations’ for machine learning approaches, and to study inter-expert variability in this domain. No prerequisites need to be met prior to sharing a project in this manner; therefore, this can be a notably quick way to commence data collection. Additionally, no expectations are placed on a research team in relation to sharing project data that has been collected through a privately launched project.

#### Public launch

‘Public launch’ refers to sharing a Zooniverse project where anyone with the project URL can contribute. In contrast to a privately launched project, a publicly launched project may be viewed by anyone, regardless of user role, and even without logging into the Zooniverse platform. Research teams may wish to adopt this approach instead of a private launch if being permissive about who can contribute does not impact study aims. As this approach requires less active project management to moderate who can contribute compared with a private launch, it can be beneficial if a large contingent of contributors are sought. Additionally, similar to a private launch, public launch is associated with no prerequisites that need to be met by the research team, and neither are there expectations associated with the sharing of data generated using this approach. Hence, a ‘project lead’ may consider adopting this approach if they would like to share their project quickly (e.g. without being subject to the review process of a full launch) or if there is some problem with sharing the data produced. Dependent on research aims, a research team may promote a public project to specific communities as opposed to the ‘general public’. However, as anyone with the URL may access the project, it should be considered whether it would be problematic for non-intended individuals to contribute, as a private launch may be more appropriate if so.

#### Full launch

‘Full launch’ refers to publicly sharing a project with the Zooniverse community. A fully launched Zooniverse project will be included on the Zooniverse projects page (https://www.zooniverse.org/projects) and shared with Zooniverse contributors via multiple communication channels (email newsletters, social media). To ensure all projects promoted by the Zooniverse in this way are a valid application of the time contributors donation to the platform, each project applying for a full launch is subject to a multi-step review process involving members of the Zooniverse research team and a self-selected community of Zooniverse contributors. This review process comprehensively assesses all project components: from the validity of applying citizen science to the specific research question being addressed, to providing study design guidance if needed. In addition to providing qualitative project feedback from the Zooniverse community, the review process provides an opportunity to generate a reasonably large amount of pilot data, which should be analysed at this stage to ensure the study design is appropriate for addressing the intended research question.

## Data analysis

After an online citizen science project has been built and shared and classifications have been made by contributors, it will be necessary to analyse the data produced. Often, multiple contributors classify each individual subject in an online citizen science project. Hence, a central component of data analysis in this context is the process of ‘aggregation’—coming to a consensus answer from multiple contributions. Beyond this core topic, we will also consider how to export and interpret project data, and the downstream applications of the data generated.

### Data export

An online citizen science project with the Zooniverse platform generates multiple types of data, including project classification data as well as data from the project ‘talk’ forums. Project data is exportable from the Project Builder interface, where it is possible to request a classification export (all, or subset by workflow), a subject export and a workflow export. These data exports can be requested at any point during the project lifecycle – it is not necessary to have launched a project or completed data collection. This permissive access to project data facilitates the iterative analysis of data, enabling analytical pipelines to be developed early in the project building process. This enables assessment of data quality (e.g. in relation to workflow version) and associated refinement of study design. The information included in the classification data export will be dependent on the tasks embedded in the project workflow, hence the most likely data outputs encountered in the context of biological volumetric data will be positional marks, circles, ovals or lines.

### Aggregation

After online citizen science project data has been collected and exported, the first step in downstream data analysis is often to implement a form of data aggregation. As outlined, this step is frequently necessary in citizen science: dependent on the aim of a project’s workflow and its associated retirement limit, multiple classifications will likely have been submitted for each individual subject; hence, it will be necessary to aggregate these individual classifications to form ‘consensus’.

The aggregation approach implemented will be dependent on a project’s workflow(s) and associated task(s) ("Workflow", Figs. 2, 3), and hence, there can be widely varying levels of complexity associated with the aggregation of data produced by different online citizen science projects. This is in part due to the inherent complexity of the data produced by different task types e.g. it is more difficult to aggregate multiple freehand lines than it is to form an approach to derive consensus from a binary question.

It should also be noted that the relative ease of aggregation can also relate to the amount of pre-existing shared code, infrastructure (e.g. Zooniverse’s ‘Caesar’ infrastructure) and the pre-existing expertise that has been shared for different task types. This is partly a product of how long different tasks have been in use and how many other teams have successfully applied that task (and made their code publicly available). For some tasks, the code generated by other research teams has been collated and made available on GitHub (https://github.com/vrooje/panoptes_analysis and https://github.com/zooniverse/Data-digging), whereas other teams have created their own repositories for code sharing (e.g. https://github.com/FrancisCrickInstitute/Etch-a-Cell-Nuclear-Envelope). Often this code is reusable between projects using the same task types, and so can be leveraged to expedite analysis. Such code and expertise sharing across projects is one of the benefits of multiple researchers using a common platform for their projects.

#### Task-specific aggregation strategies

In the online citizen science projects led by the ‘Etch A Cell’ and ‘Science Scribbler’ teams, the question and drawing tasks were frequently used ("Workflow"). Aggregating data produced by the question task is relatively straightforward, as responses can be combined using a simple majority voting strategy, and as this task has been used extensively on the Zooniverse platform, much pre-existing code and infrastructure exists to support analysing the data generated by this task type. For drawing tasks, significantly different aggregation approaches will be required dependent on the specific drawing task type used for annotation (e.g. ‘ellipse’, ‘point’, ‘freehand line’, etc.). Distinct approaches will be required when annotating data through outlining a feature of interest with a freehand line, versus annotating features with a single mark using a point.

To give an overview, when outlining an object with a freehand line drawing task type, substantial overlap would be expected between (the area masks of) correct classifications, and therefore it is possible to compare classifications on a pixel-by-pixel basis to identify the areas with most and least consensus. Outliers may then be identified and removed through an arbitrary cut-off, comparison with an expert annotation (if available) or through visual inspection of low consensus areas, and remaining annotations may then be aggregated into a consensus segmentation.

Conversely, when annotating a feature of interest with the point drawing task type (e.g. to annotate a feature with a single central mark), overlap between these single points is unlikely. Hence, aggregation of points requires clustering in space. Because the data and features are 3D, clustering in all directions is recommended. A combination of outlier removal using a density based clustering method and then averaging the locations is an effective method of aggregation. Additional review by experts or contributors through a cleaning task can be a useful intermediate step within the clustering process to help identify outliers in crowded environments where objects can be stacked on top of one another.

#### Aggregation tools developed by Etch A Cell, Science Scribbler teams

As part of the ‘Science Scribbler: Virus Factory’ project, an extension to the SuRVoS2 volumetric segmentation package (https://github.com/DiamondLightSource/SuRVoS2) (Pennington et al. 2022) was made to assist the process of data aggregation. This consisted of support for ‘objects’ or locations with an associated classification, a format which fits the data to be aggregated in a project such as ‘Science Scribbler: Virus Factory’. A simple CSV format allows importing 3D coordinates and their classifications and displaying colour-coded points over a volumetric image. The CSV can be viewed in a table and the image zooms to a point when the row containing that point is clicked on. Several other plugins for SuRVoS2 were made that support ‘objects’. The spatial clustering plugin allows DBSCAN (Ester et al. 1996) and HDBSCAN (McInnes et al. 2017) clustering of points. The rasterize points plugin uses ‘objects’ as input, allowing a crowd-sourced workflow to be quickly turned into mask annotation. Finally, the label analyzer and label splitter tool and find connected components tool all output ‘objects’, allowing crowd-sourced workflows to be compared with connected component analysis from a segmentation. By combining these tools for geometric data processing with tools for volumetric image segmentation, SuRVoS2 allows image analysis using crowdsourced annotations to be performed in a single GUI tool.

To give a specific example of aggregation for a drawing task, in the initial ‘Etch A Cell’ project (Spiers et al. 2021), a ‘freehand line’ drawing tool was used within the project workflow, and volunteers were asked to draw around the NE. Because this was the first time this tool had been used, a novel aggregation algorithm, contour regression by interior averages (CRIA), was developed. Briefly, this algorithm involved the formation of closed loops from volunteer segmentations for each subject, generation of interior areas from these closed loops, overlaying of areas to form a height map and deriving consensus through taking a mean ‘height’ level: the consensus segmentation for each slice surrounded all the interior areas where half or more volunteer segmentations were in agreement. This algorithm has been shared for re-use by the research community (https://github.com/FrancisCrickInstitute/Etch-a-Cell-Nuclear-Envelope).

Although aggregation methods in this domain remain diverse due to the variety of workflows being implemented, we recommend the following general principles; where possible, the aggregation algorithms developed should not require active researcher involvement (e.g. manually removing poor data or seeding the algorithm etc.). Full automation and associated removal of manual intervention not only speeds the analytical pipeline, it also removes opportunity for bias – increasing the objectivity and reproducibility of the aggregation algorithm. We recommend the aggregation approach make as full use of volunteer annotations as possible, and we recommend all code developed in this context be shared for re-use by the research community ("Project impacts").

### Applications

All project building, data collection and data analysis decisions will be informed by the intended application of the classification of data sought—the research question (Fig. 1). Most often, aggregated data generated by an online citizen science project will be used to address a specific research question, such as what changes can be observed in the morphology of the endoplasmic reticulum when a cell divides (‘Etch A Cell - ER’) or how are different virus stages spatially located relative to other virus stages or cellular components (‘Science Scribbler-Virus Factory’). In addition, the scale of the generated data made possible by online citizen science frequently allows the additional application of training machine learning algorithms for the automated analysis of further datasets. We consider below the application of aggregated data as an input for machine learning models for object detection, segmentation and classification, although other models, such as point cloud segmentation or image understanding and captioning, could be useful.

#### Machine learning

For machine learning-based object detection, models are usually trained on geometric annotations, such as bounding boxes or point locations. Bounding boxes provide a set of 2D or 3D coordinates that define a rectangular volume, where the extent of the bounding box indicates the extent of the object in the image. Aggregated data from a Zooniverse workflow with a rectangular drawing task may be used as input, or if the object of interest is of a consistent size and shape, centres/centroids may be used to create appropriate bounding boxes. In contrast, machine learning image segmentation models are usually trained to predict masks either using masks as training data, or annotations that indicate the assignment of a region to a particular segmentation class. Aggregated outputs from a Zooniverse workflow with a drawing task where contributors are asked to outline or fill in objects of interest can be used in this case (Spiers et al. 2021). Classification models can be applied to the outputs of either object detection or segmentation models using classifications procured from a Zooniverse question-based task as input training data.

The ‘Etch A Cell’ project provides an example of aggregated contributor annotations being used to train machine learning models for segmentation of the NE and their application to unseen cells (Spiers et al. 2021). While the ‘Science Scribbler: Virus Factory’ project provides an example of all three machine learning strategies based on aggregated contributor annotations, used in concert to detect, segment and classify viruses inside of a cell.

#### Producing high quality training data

Machine learning models generally perform best when the provided training data is as unambiguous as possible and strategies for reducing noise in training data are essential for training useful models. Great care must be taken when curating contributor data for the purpose of training machine learning models, and to this end, both contributor-based and automated strategies to ensure data cleanliness should be considered. As a general approach, after an initial workflow that produces locations, a follow-up workflow in which contributors may be instructed to stringently classify previous locations was found to significantly improve annotation quality. This approach was used in ‘Science Scribbler: Virus Factory’ and more recently in ‘Science Scribbler: Placenta Profiles’. Automated approaches that filter annotations on the basis of some measure of annotator performance or image difficulty may be useful for reducing outliers (Branson et al. 2017). In addition, automated approaches for outlier removal may be helpful. For example, in ‘Science Scribbler: Virus Factory’, HDBSCAN (McInnes et al. 2017; Campello et al. 2013) clustering and outlier removal was used for processing the set of locations provided by an initial crowdsourcing workflow. This worked well as the locations provided by the initial workflow were often clustered closely together around the centroid of the object of interest and outlier locations appeared to often correspond to unique errors on the part of individual contributors.

#### Machine learning outputs as an input to citizen science projects

While here we have focussed on how online citizen science data may be used as a machine learning input, it should be noted that, reciprocally, machine learning outputs may be used as an input to online citizen science. Subjects may be presented to contributors that contain information generated by pre-existing machine learning models. For example, in ‘Etch A Cell - VR’, the predictive model generated by ‘Etch A Cell’, (Spiers et al. 2021), was used to pre-segment the NE in the data for this project, and subjects were generated with this feature already segmented to provide additional context information to the contributors. In the second workflow of ‘Science Scribbler: Virus Factory’ (Fig. 3), locations that had been processed through a spatial clustering algorithm were presented to contributors as animated GIFs so they could inspect carefully and classify them.

## Project management

The fourth and final core stage of the Zooniverse project lifecycle is ‘Project Management’. Although we present recommendations in relation to this component last, it should be noted that many of these best practices are pertinent throughout project lifecycle. Three broad themes shall be considered in relation to managing a project effectively; engagement, efficiency and impact.

### Project engagement

It is recommended that the project lead and their collaborators take a proactive approach to engaging with the community of individuals contributing to their online citizen science project. First, it is important that the research team, who are directly benefiting from time voluntarily donated to their project, reciprocate this effort by sharing further information relevant to their project. Beyond providing a mechanism to reciprocally engage with project contributors, such communication can help build an active community, therefore aiding faster completion of data annotation. Proactive engagement with project contributors may also advance project aims through providing a mechanism for receiving, and giving, useful feedback—enabling the research team to garner insight that may improve project design and clarify any issues unclear to project contributors. It is because of the multifaceted importance of such proactive communication that it is considered a requirement of projects fully launched on the Zooniverse platform ("Launch"); however, the principles of community engagement remain relevant to projects launched either privately or publicly.

#### Talk boards

Available to all projects is the talk discussion board tool. This feature supports two core functions—it allows contributors to comment on subjects they have analysed, and provides a space for project leads and contributors to interact. Commonly encountered comments on talk boards include discussion relating to individual subjects (e.g. ‘Is this a mitochondrion?’), broader scientific questions (e.g. ‘What kind of cells are being visualized?’) and technical issues (e.g. ‘What happens if I’ve pressed the wrong button?’). We recommend questions are responded to quickly, and that repeatedly encountered comments or issues are addressed through study design changes or by updating project materials, such as the tutorial or frequently asked questions ("Training").

#### Internal communications

For projects fully launched on the Zooniverse platform ("Launch"), a range of additional communication modes are available. Project leads can write newsletters which can be shared to a mailing list of registered Zooniverse contributors who have contributed and are subscribed to receive emails. Newsletter content can include any project-relevant information e.g. announcing a new data upload, project milestone or project results, and can provide an opportunity to re-engage previously active members of the community. Project leads of fully launched projects may also work with the Zooniverse team to create and share content through two blog forums, The Daily Zooniverse (https://daily.zooniverse.org/) and The Zooniverse Blog (https://blog.zooniverse.org/). Each adopts a slightly different format, with The Daily Zooniverse typically being a short-format blog post centred around a featured image, whereas longer posts are published via The Zooniverse Blog. Content for either blog can be diverse, provided it is project relevant, from unusual project images to interviews with project collaborators and contributors. Posts to either blog are automatically shared to Zooniverse social media accounts, including Twitter and Facebook.

#### External communications

Beyond the communication mechanisms available through the Zooniverse platform, a project lead may take other approaches to proactively engage with contributors. Additional approaches include, but are not limited to, promoting online citizen science projects via social media, creating project-specific blogs and running email lists. Some of these mechanisms have been applied in the context of the ‘Etch A Cell’ and ‘Science Scribbler’ projects, e.g. the ‘Etch A Cell’ team have shared much project content through social media.

#### Outreach events

Finally, as online citizen science projects frequently represent a microcosm of the research process and are designed in such a manner to enable the broadest possible audience to contribute, they often provide a natural centre piece for outreach events. Both ‘Etch A Cell’ and ‘Science Scribbler’ projects have been showcased in events and exhibitions, including the Natural History Museum Lates series (https://www.nhm.ac.uk/events/lates.html) and London Tech Week (https://www.crick.ac.uk/news/2018-06-11-london-tech-week-launched-at-the-crick). Similarly, online citizen science projects often translate effectively into educational settings (Bonney et al. 2009), e.g. through the recently implemented ‘scribbling for science in schools: taking authentic research into local schools with the Zooniverse’ programme, the ‘Science Scribbler: Virus Factory’ project was used as the focus of a series of 3-hour long educational workshops for primary school pupils in the UK. These workshops allowed the students to engage with the research team, contribute to the citizen science project, and learn about virus biology and data analysis concepts.

### Project efficiency

On the Zooniverse platform, there are multiple features available that can be applied to improve project efficiency and the quality of the output annotations. These features including mechanisms for providing feedback to contributors, educational mini-courses, and the early retirement of subjects. Such features have the broad aim of reducing the number of classifications a project requires, increasing the speed of classification and improving the quality of the classifications collected.

Two of these features have been applied within ‘Science Scribbler’ projects; the ‘feedback system’ and the educational ‘mini course’. The ‘feedback system’ provides a mechanism for giving contributors feedback on the outcomes of their work (e.g. whether their classifications are correct or incorrect). To use this feature, a number of subjects were classified by the experts prior to project launch, then added to the project as ‘training subjects’ and presented to the contributors at a designated frequency. Usually, ‘training subjects’ are shown more frequently when a contributor starts working on the project and the frequency gradually decreases as they continue with the same project. Each time a ‘training subject’ is presented, a feedback message is given. In all cases, the training subjects should be carefully chosen, with consideration given to the introduction of bias from the feedback itself. If difficult subjects, or edge cases, are chosen, bias towards confirming or rejecting a classification could be introduced, or contributors could be discouraged by perceived ‘wrong’ answers. By providing instant feedback, contributors can improve their knowledge about the subjects, which can help them make a better decision in the subsequent subjects. Hence, the ‘feedback system’ is a useful tool not only to motivate but also to train the contributors.

The ‘mini course’ is another feature that can be used to enrich the contributor experience by supplying further information related to the project. In contrast to the ‘feedback system’, the ‘mini course’ is not associated with the performance of the contributors and not linked to individual subjects. Instead, it provides a means of giving additional non-task-related communication with the contributors at a defined interval. The ‘Science Scribbler’ team has used mini courses to provide ‘fun facts’ about the science topic as part of the ‘Science Scribbler: Placenta Profiles’ project. By sharing further information, the contributors gain greater insight into the research they are taking part in, and previous research has indicated that these communications improve motivation and engagement of the contributors (de Vries et al. 2019).

Early retirement of subjects with high consensus can increase project efficiency. This feature allows the research team to set a consensus value below the defined retirement limit ("Workflow"), which will trigger the early removal of the subject from the pool. For example, if a subject with retirement limit of five is classified identically by three contributors, the majority consensus will have been reached, and the final two classifications will not alter the outcome. By retiring this subject early, the final two contributors can be presented a different subject, thus reducing the total required contributor effort, and increasing the meaningful contribution by each volunteer. Although this feature has not yet been implemented by the ‘Etch A Cell’ or ‘Science Scribbler’ teams, it is being considered for future projects.

### Project impacts

**Table 3:** Recommended approaches for sharing project outputs. Online citizen science projects for the analysis of biological volumetric data can result in a diverse selection of project outputs that should be openly shared in a timely manner

Project output example	Recommendations	Possible venues
Raw image data e.g., www.ebi.ac.uk/pdbe/emdb/empiar/entry/10094	Large, complex data sets should be deposited in a public image archive to enable access.	$\bullet$ Electron microscopy public image archive (EMPIAR; www.ebi.ac.uk/pdbe/emdb/empiar) $\bullet$ Image Data Resource (IDR, idr.openmicroscopy.org) $\bullet$ BioImage Archive (www.ebi.ac.uk/bioimage-archive) $\bullet$ BioStudies database (www.ebi.ac.uk/biostudies) $\bullet$ Cell centered database (library.ucsd.edu/dc/collection/bb5940732k) $\bullet$ Nanotomy (www.nanotomy.org) $\bullet$ OpenOrganelle (openorganelle.janelia.org) $\bullet$ WormAtlas (www.wormatlas.org/index.html)
Raw contributor data e.g., www.ebi.ac.uk/biostudies/files/S-BSST448/classifications.tar.gz e.g., www.ebi.ac.uk/biostudies/files/S-BSST448/Expert	Sensitive fields should be removed from the classification export prior to sharing. Sharing contributions as a spreadsheet rather than binary masks will reduce the digital footprint. A disclaimer should be provided if the data may contain offensive classifications. Relevant data contributed by experts should also be shared.	$\bullet$ GitHub (www.github.com) $\bullet$ Biostudies (www.ebi.ac.uk/biostudies)
Aggregated contributor data e.g., www.ebi.ac.uk/biostudies/files/S-BSST448/Aggregations	The mode of data aggregation should be carefully communicated in conjunction with aggregated contributor data. Data may be shared within a spreadsheet via a binary mark.	$\bullet$ GitHub (www.github.com) $\bullet$ Biostudies (www.ebi.ac.uk/biostudies)
Aggregation code e.g. www.github.com/FrancisCrickInstitute/Etch-a-Cell-Nuclear-Envelope	Aggregation algorithms should be shared for re-use.	$\bullet$ GitHub (www.github.com)
Machine learning code and data e.g. www.github.com/FrancisCrickInstitute/Etch-a-Cell-Nuclear-Envelope e.g. www.ebi.ac.uk/biostudies/files/S-BSST448/Predictions	The accessibility of any shared code for the intended end-user should be considered.	$\bullet$ GitHub (www.github.com) $\bullet$ BioImage Model Zoo (www.bioimage.io) $\bullet$ ZeroCostDL4Mic (www.github.com/HenriquesLab/ZeroCostDL4Mic) $\bullet$ Model Zoo (www.modelzoo.co) $\bullet$ Jupyter notebooks
Microscopy protocols	Version control should be carefully applied to ensure precise information is captured for each iteration of an experiment.	$\bullet$ Protocols.io (www.protocols.io)

An online citizen science project can result in a range of different outputs (Table 3) which may be useful to a variety of research communities, including domain specific research scientists, citizen science researchers, social scientists, machine learning specialists and computer scientists. In the case of a fully launched Zooniverse project, these outputs should be shared as openly as possible and in a timely manner. For private and public projects, the following section should be seen as a recommendation.

#### Biological volumetric data

The first output of online citizen science projects in the domain of biological volumetric image analysis is typically the raw ‘input’ data that is served to the project contributors ("Data"). In the case of ‘Etch A Cell’ and ‘Science Scribbler’, these are images acquired using high-end imaging platforms - electron and X-ray microscopes. These images may be saved in formats that are inaccessible to the lay-person and are often too large to open in non-scientific software. The recommended route to share these large, complex datasets is to deposit them in a public image archive (Table 3). The European Molecular Biology Laboratory European Bioinformatics Institute (EMBL EBI) provides the Electron Microscopy Public Image Archive (EMPIAR) for this purpose (Iudin et al. 2022). The archive can ingest images and associated metadata from electron microscopes (transmission, scanning and volume), as well as X-ray images of cells and tissue acquired at synchrotrons. Similarly, the Image Data Resource (IDR) fulfils the same function for published images of cells and tissues, mainly acquired using light microscopy, but also from other imaging modalities (Tarkowska et al. 2017). These resources will be supported in the future by the BioImage Archive (Hartley et al. 2022), and link to resources such as the BioStudies database (Sarkans et al. 2018) to ensure that all images are linked to relevant project-level metadata. Alternative image archives include the Cell Centered Database (Martone et al. 2003), Nanotomy (de Boer et al. 2020), OpenOrganelle (Xu et al. 2021) and WormAtlas (White et al. 1986). Communities re-using these data will include biologists mining rich image datasets for new insights, software developers looking for test image datasets and the general public interested in biology, microscopy and the research outputs of public funding.

#### Raw classification data

The second output from an online citizen science project is the raw project data generated by contributors. Raw contributor data from the ‘classification export’ ("Data export"), should be shared for re-use. The ‘classification export’ contains, amongst other information, the co-ordinates of the annotations, which can be converted into a binary mask in the case of direct annotations or centre points, ovals or bounding boxes as appropriate. These data may be shared as a spreadsheet to different open access environments like GitHub or BioStudies (Table 3). Prior to sharing this raw contributor data, the project lead should consider what fields from the classification export are included, as some information may be sensitive. When sharing raw project data, the ‘Etch A Cell’ team removed usernames and hashed IP addresses, to ensure contributor privacy. Raw contributor data may also be shared as binary masks; however, due to the potentially large number of contributor classifications, this can represent a very large digital footprint, and so may not be advisable as an approach to sharing raw contributor data. As project data may occasionally contain offensive words or drawings, research teams may wish to provide a disclaimer alongside their raw data upload. Finally, any raw contributions created specifically by experts in relation to the research project should also be shared.

#### Aggregation algorithms and data

The third output from an online citizen science project is ‘aggregated data’ ("Aggregation") and any associated novel aggregation algorithms, e.g. a novel algorithm to aggregate the NE segmentations submitted to ‘Etch A Cell’ was developed and shared (Spiers et al. 2021). New insights and approaches are best shared through open access scientific publications, with associated code deposited into GitHub, and aggregated data shared either as binary masks via public image archives or as spreadsheets through GitHub or BioStudies (Table 3).

#### Machine learning algorithms and predictions

Beyond being used to address specific research questions, the data from an online citizen science project can be used to train machine learning algorithms to recognise structures in the raw data and additional naïve data, with the aim of removing the bottleneck of expert manual annotation and maximally leveraging the contributor contributions to automate processes and speed up discovery science. Hence, the fourth output type typical of an online citizen science project is novel machine learning algorithms and their predictions ("Application"). Outputs in this case include new technical insights and approaches, code and additional binary masks, all of which can be shared as above. In addition, automated approaches, such as deep learning and other AI methods, may output probability maps that could be stored as floating point or greyscale images that permit further post-processing steps and models that could be deposited in open resources such as GitHub, BioImage Model Zoo (Ouyang et al. 2022), ZeroCostDL4Mic (von Chamier et al. 2021), Model Zoo or as Jupyter notebooks (Kluyver et al. 2016) to ensure they are easily accessible (Table 3). A key concept here is ensuring easy access and usability for the end-user, who will usually be a biologist or microscopist, and who may have limited training in coding and non-GUIs. For this reason, if it is possible, the approach should also be integrated into a commonly used, easily accessible software tool, such as ImageJ (Schindelin et al. 2015; Rueden et al. 2017), FIJI (Schindelin et al. 2012) or napari (Sofroniew et al. 2022).

#### Protocols

Finally, the fifth major output will be a range of protocols that have been used during the course of the project, from those used to grow cells or manipulate genes, to sample preparation protocols for microscopy, to workflows for pre- and post-processing of image data, algorithm design and deep learning methods. These protocols should be openly shared in a way that allows faithful reproduction of the work, through open access journals or repositories such as GitHub and platforms like protocols.io (Table 3).

## Discussion

Generation of biological volumetric data is rapidly gaining momentum, yet data analysis strategies are failing to keep-pace, meaning human effort remains a critical component of many data analysis pipelines. Research teams are increasingly turning to untraditional approaches to gather human annotations, including citizen science. Here, we have collated the experience and insights gained by researchers leading multiple citizen science projects in this arena to create this primer for teams wishing to create similar projects. Here we discuss the core considerations identified in relation to our four online citizen science project lifecycle stages: project building, data collection, data analysis and project management. We reflect on limitations of the approaches discussed and highlight opportunities for the extension and development of work in this domain.

All ‘Science Scribbler’ and ‘Etch A Cell’ projects to date have been developed and launched with the Zooniverse project builder online interface for citizen science project building. Data preparation and presentation were identified as central components of this lifecycle stage. Data need to meet the technical requirements of the platform and should be optimised to support the effective completion of the project tasks. There is much a research team can adjust to improve data presentation; variables such as selecting high quality data, selectively labelling the features of interest to make them more identifiable and isolating ROIs to focus effort upon, etc.

Though individual data files are size-limited, this need not limit the overall size of the project dataset. By combining appropriate FOV selections and tiling strategies, manageable workflows can be created for large, high resolution datasets without loss of annotation quality. However, projects with a seemingly insurmountable volume of work may struggle to recruit and retain contributors. Additionally, given that publicly launched projects must be shown to be an appropriate use of volunteer effort, research teams should aim to design projects that effectively answer the research question whilst minimising contributor effort. Not only will reducing overall project size and maximising volunteer engagement expedite the timeline to completion, but this approach naturally lends itself to designing projects with machine learning applications in mind. In this way, the contributor effort can be maximally leveraged, not only to produce a single annotated dataset, but to provide the basis for automated annotation of future unseen data.

### An accessible tool for the visualization of volumetric data

Appropriate display of volumetric data to contributors is clearly a critical consideration in the context of the projects presented in this manuscript. Although the Zooniverse currently only offers limited functionality for the presentation of 3D data, of the data types available, image, video and GIF data types have proven suitable for the display of biological volumetric data for ‘Science Scribbler’ and ‘Etch A Cell’ project collections. The high quality of annotations submitted to projects to date (Spiers et al. 2021) indicate that current modes of data presentation are fit for purpose; however, clearer presentation of 3D data would likely improve contributor experience, engagement and data accuracy. The platform could also benefit from an expansion of supported imaging data types, by integration with tools, such as Bio-Formats, to allow for the presentation of a wider variety of microscopy sources (Moore et al. 2015).

Improved visualisation of volumetric data remains a significant opportunity for future development of the Zooniverse platform, particularly as this would bring the contributor experience more in-line with that of professional researchers, who commonly flip through the 3D space to glean additional contextual information when deciding how to annotate. A more intuitive display of the 3D data would include features such as interactive display and control of other data planes for isotropic data, and indication to the contributor where within the overall volume they are annotating. Advancing data presentation options will likely become increasingly important as novel projects, with more complex analytical needs, are conceived and developed, although benefits associated with any modifications would need to be validated with testing, particularly as a more complex interface may discourage some contributors from participating.

Developing customised interfaces and bespoke tools specific to a project’s aims is more straightforward when building a project independently of a platform with generic project building solutions. This benefit to taking an independent approach can be illustrated by the great success, and beautiful design, of stand-alone online citizen science projects for the analysis of biological volumetric data such as Eyewire (Kim et al. 2014). However, creating such projects can be costly in time and resources. Conversely, although building a project using the generic solutions provided by pre-existing platforms can reduce study design options, taking this approach can significantly reduce project build time and cost amongst other benefits, including connection to an established community of potential contributors, researchers and experienced web developers. The cost: benefit relationship of different project building solutions is nuanced and will be largely project dependent, relating to variables such as budget, expertise, research needs and desired timelines. For the projects developed by the ‘Etch A Cell’ and ‘Science Scribbler’ teams, the project builder solution provided by the Zooniverse was found to be suitable for project development.

### Designing for your crowd

When building a project, a research team must consider who their project is going to be shared with and develop their project according to the anticipated needs of this intended community. A critical consideration is whether a project will be made publicly accessible to all, or whether it will be shared privately with a defined community, such as professional researchers, a patient group or students. This decision will be informed by project needs, such as speed of analysis desired, the amount of data to be processed, and whether specific skills are needed for annotation. If a project is to be publicly accessible, building with an established platform offers an advantage through providing an opportunity to connect with a large pre-existing community of contributors who are often already familiar with the concept of citizen science and the tasks commonly involved, and who may even have experience working on similar datasets with similar tools, and this will become increasingly true as the number of projects involving biological volumetric data analysis on the Zooniverse continues to grow. However, public launch on the Zooniverse is associated with pre-requisites, including passing review with team members and contributors, and expectations are placed on the eventual public sharing of project classification data. Clearly these constraints do not exist for projects independently built. Yet, there are arguably advantages to the imposition of requirements and standards on the building of projects to be shared with the public. Expertise and knowledge are shared through the review process by members of the Zooniverse team and contributor community, leading to the improvement of projects.

We anticipate that this domain will continue to be dominated by open, publicly accessible projects, as this enables working with the largest community, and thus maximises project efficiency and impact. Yet, as this field continues to develop, there may be greater opportunity, and need, to connect with specific communities. As the research needs of online citizen science projects in this domain become more refined and focussed, there may be scope to identify groups of contributors with specific skill sets well-suited to particular tasks, e.g. there may be groups of contributors who are expert at identifying particular organelle classes, or groups who are particularly expert at segmenting. It is possible to conceive projects in this domain where a contributor’s skills are identified, and then their expertise leveraged. This would benefit both project efficiency and likely support contributor engagement. The accessibility of online citizen science projects may also allow standardization of viewing and annotating biological volumetric data across research communities, particularly as researchers without access to traditional drawing tablets or expensive software will be able to take part, enabling analysis to be distributed across larger groups. Further, because the time investment is small relative to annotation of the full dataset, researchers can easily perform the tasks on a small number of subjects in between other activities, rather than setting aside hours or days to annotate the entire dataset.

The crowd connected with will likely impact project workflow design—another key consideration in online citizen science project building. Often the information needed to answer the research question can be gathered in multiple ways. As such, workflow design choices should be made with the following guiding principle in mind: workflows should be designed to collect the needed data in the simplest way for the contributors. To date a small number of task types have been applied to the analysis of biological volumetric data on the Zooniverse platform. ‘Drawing’ and ‘question’ tasks have dominated. However, the dominance of these task types in the workflows of ‘Etch A Cell’ and ‘Science Scribbler’ projects does not preclude other task types from being potentially useful in this context. For instance, as our ability to automatically annotate biological volumetric data becomes more advanced, it may be that ‘survey’ or ‘text’ tasks become more useful to classify multiple distinct organelle classes in a single subject.

### Consider strategies for tackling data multiplication

Following project building and data collection, research teams will need to analyse the data generated. It is often overlooked that citizen science projects are often data transformation pipelines rather than data analysis pipelines. Generally, image data is the input, contributors annotate these, and the outputs are the locations or descriptions of objects or features of interest within the dataset. The image data has been transformed to location or description data; however, it has not yet been analysed. Relatedly, it is often overlooked that citizen science workflows tend to multiply the data. Multiple contributors assess each subject, and multiple objects may be annotated in each subject. Multiple tasks or workflows may be necessary to ensure the production of high-quality annotations. Together, this can create a far larger amount of data than if one expert did the analysis a single time—one of the greatest challenges in performing research in conjunction with citizen scientists is to make sense of the multiple classifications submitted. Careful thought needs to be given to how project outputs will be analysed, with consideration given to outlier detection and aggregation strategies. Building on a platform where others have used the same tools means aggregation code can be re-used between projects. However, translation of approaches between similar projects can sometimes require careful consideration, for example, aggregation can sometimes be optimised to improve results for different organelle classes, with incorporation of pre- and post-processing steps to improve performance. As research teams continue to work in this arena, we anticipate the consolidation and streamlining of these analytical tools for reuse by other teams.

### The potential for reduced bias and serendipitous discovery

Although citizen science often involves multiple individuals analysing each subject, creating a need for data aggregation and consensus forming, there are several subtle advantages to working in this capacity. Asking multiple individuals to classify the same subject may reduce the impact of inter-individual variation and bias commonly seen in this domain, leading to a more ‘objective’ analysis and reducing the impact of, say, one expert who may be biased in some capacity in their segmentation. Creating as objective as possible ground-truth data will become ever more critical when it is used to train automated algorithms for high-throughput analyses. Lack of prior knowledge and bias in an annotation task may also result in the identification of visually different variants of an organelle class that may be overlooked by an expert annotator. This may lead to the identification of structurally distinct, but biologically important, organelle variants. Beyond this, there will likely be biologically interesting phenomenon in regions of disagreement – adding further value to having multiple individuals annotate the same regions in biological volumetric data. We anticipate that the approach of working with multiple individuals to analyse a single dataset will prove useful even just with experts, hence, the analytical approaches developed through citizen science, including retirement, aggregation and consensus, will likely find use in other contexts.

### Creating an open and collaborative future for citizen-powered biological image analysis

Making the data, code and machine learning models generated by citizen science projects available for re-use was identified as an important component of Project Management. Ensuring prior work is openly discoverable and easily reusable is not only a reasonable expectation to place on outputs generated through contributor effort, but it will also help advance data analysis in this arena. Repositories for data, such as EMPIAR, and machine learning model repositories, such as BioImage Zoo, will play an important role in the consolidation, sharing and re-use of the outputs generated. It may be that in the future projects will involve direct pulling and pushing of data and models to these repositories, to be viewed, re-used and corrected in real-time. As this shift to review and correction occurs, new tools to allow easy comparison of multiple annotations or predictions in the same area of a dataset will be needed. This could be used for one-to-one comparisons or with groups of data to identify serendipitous features, or outliers for removal. With the inclusion of the ability to provide corrections to annotations, or append additional annotations to use during a re-training step to correct predictions, an iterative human-AI loop could created. This process will also lead to the need and opportunity to develop collaborative or collective segmentation strategies to ensure consensus is built in ways which give accurate outcomes, yet still allow for serendipity, even in situations where a 'ground truth' segmentation is unavailable.

### Other ways to crowdsource data analysis

Throughout this manuscript we have limited our scope to discussing projects generated with the Zooniverse online citizen science platform; hence, it is important to reiterate that other options exist for creating projects to connect with online communities for the analysis of data. There are an increasing number of established crowdsourcing platforms that enable completion of micro-tasks through the engagement of large communities. These platforms include Amazon Mechanical Turk (https://www.mturk.com/), Wazoku (https://www.wazoku.com), Openideo (https://www.openideo.com/), Quantius (Hughes et al. 2018) and WebKnossos (Boergens et al. 2017), amongst others. While such pay-for-service options have a clear benefit of being able to facilitate the collection of a large amount of data in a short amount of time, it should be acknowledged that they have several disadvantages that can preclude their useful application. For example, the cost to generate data with pay-for-services may immediately rule this option out. If within budget, a research team considering this approach will need to be mindful of possible unanticipated consequences of using paid-for crowdsourcing platforms. For example, providing a financial incentive can result in crowds contributing less to a task that they would otherwise be intrinsically motivated to complete (Frey and Jegen 2001). Creating further challenges, a significant amount of the data generated by platforms such as MTurk can be ‘suspicious’, generated by bots, non-serious respondents and even individuals deliberately misrepresenting themselves (Ahler et al. 2019; Webb and Tangney 2022). As discussed, another project building option is to create a stand-alone, fully customised project for a specific task, e.g. ‘Eyewire’ (Kim et al. 2014). While independently building a stand-alone project confers great flexibility to create a bespoke design for a specific use-case, such ground-up construction of a full project requires significant time, expertise and resources. Conversely, although a platform with generic project building functionalities such as the Zooniverse constrains building possibilities to the features available, many benefits are conferred, such as having connection to a community of researchers, developers and volunteers with pre-existing expertise.

### Final thoughts

The field of volumetric biological imaging will continue to grow, and alongside this growth more accurate and efficient machine learning strategies for data analysis will become widely available. Already, work is underway to create and publicly archive annotations and segmentations for re-use as training datasets and to explore the transferability of annotations across datasets. Eventually, most data produced will be annotated using semi-automatic or automatic pipelines, shifting the analytical bottleneck to the validation, review and correction stage of the workflow. This phase will require additional tools and task modalities to enable efficient comparison and review of annotations. As more research teams continue to move into this arena, we will see further examples of how online citizen science can be meaningfully and usefully applied to biological volumetric data analysis.
